# Growth Modeling for Quantitative, Spatially Resolved Geographic Atrophy Lesion Kinetics

**DOI:** 10.1167/tvst.10.7.26

**Published:** 2021-06-22

**Authors:** Eric M. Moult, Yunchan Hwang, Yingying Shi, Liang Wang, Siyu Chen, Nadia K. Waheed, Giovanni Gregori, Philip J. Rosenfeld, James G. Fujimoto

**Affiliations:** 1 Department of Electrical Engineering and Computer Science, Research Laboratory of Electronics, Massachusetts Institute of Technology, Cambridge, Massachusetts, USA; 2 Health Sciences and Technology, Harvard & Massachusetts Institute of Technology, Cambridge, Massachusetts, USA; 3 Department of Ophthalmology, Bascom Palmer Eye Institute, University of Miami Miller School of Medicine, Miami, Florida, USA; 4 New England Eye Center, Tufts Medical Center, Boston, Massachusetts, USA

**Keywords:** geographic atrophy, cRORA, growth rates, growth modeling, lesion kinetics

## Abstract

**Purpose:**

To demonstrate the applicability of a growth modeling framework for quantifying spatial variations in geographic atrophy (GA) lesion kinetics.

**Methods:**

Thirty-eight eyes from 27 patients with GA secondary to age-related macular degeneration were imaged with a commercial swept source optical coherence tomography instrument at two visits separated by 1 year. Local GA growth rates were computed at 6-µm intervals along each lesion margin using a previously described growth model. Corresponding margin eccentricities, margin angles, and growth angles were also computed. The average GA growth rates conditioned on margin eccentricity, margin angle, growth angle, and fundus position were estimated via kernel regression.

**Results:**

A total of 88,356 GA margin points were analyzed. The average GA growth rates exhibited a hill-shaped dependency on eccentricity, being highest in the 0.5 mm to 1.6 mm range and lower on either side of that range. Average growth rates were also found to be higher for growth trajectories oriented away from (smaller growth angle), rather than toward (larger growth angle), the foveal center. The dependency of average growth rate on margin angle was less pronounced, although lesion segments in the superior and nasal aspects tended to grow faster.

**Conclusions:**

Our proposed growth modeling framework seems to be well-suited for generating accurate, spatially resolved GA growth rate atlases and should be confirmed on larger datasets.

**Translational Relevance:**

Our proposed growth modeling framework may enable more accurate measurements of spatial variations in GA growth rates.

## Introduction

Geographic atrophy (GA), also termed complete retinal pigment epithelium and outer retinal atrophy,^1^ is a late stage of age-related macular degeneration characterized by contiguous regions of photoreceptor, retinal pigment epithelium, and choriocapillaris atrophy.^2^^–^^5^ Currently, there are no approved treatments to stop or slow lesion growth and GA causes progressive vision loss.^6^ Importantly, GA growth rates exhibit variability at multiple spatial scales^7^: on a global (whole lesion) scale, there is eye-to-eye and patient-to-patient variability in GA growth rates; and, on a local (margin segment) scale, there is variability in the growth rates along different lesion segments.

At present, variations in GA growth rates remain incompletely understood, although various explanatory covariates have been proposed, including lesion geometry,^8^^,^^9^ choriocapillaris status,^10^^–^^12^ and fundus autofluorescence patterns.^13^ Studies investigating GA lesion kinetics as a function of lesion position have found that growth rates exhibit a hill-shaped dependency on eccentricity, growing slower in the fovea and outer macula, and faster in the perifovea and parafovea.^14^^–^^16^ In particular, the phenomenon of foveal sparing, whereby some GA lesions preferentially expand around and/or away from, rather than toward, the fovea, is well-documented.^7^^,^^17^ Prior studies investigating spatial variations in GA growth rates have tended to use global (i.e., not spatially resolved) growth rate measurements applied to grids (grid-based),^14^^,^^15^ or, in some cases, to particular lesion configurations (configuration-based)^17^—for example, lesion configurations that include residual foveal islands. However, in a local approach, Uji et al.^18^ used Euclidean distance maps (distance-based) to study spatial variations in GA growth rates.

Our group has recently introduced a GA growth modeling framework that enables the estimation of local GA growth rate measurements.^19^ Because this framework allows for spatially resolved measurements along the en face lesion margin, it is naturally suited for directly quantifying spatial variations in GA growth rates. In this study, we demonstrate the applicability of our growth modeling framework for spatially resolved GA growth rate measurements in a pilot study of 38 eyes having 1-year follow-up intervals. We believe that this application of our model is important because quantifying GA growth variability may improve our understanding of GA pathogenesis, decrease measured variations in GA therapeutic trials, and refine predictive GA growth models.

## Methods

**Figure 1.**
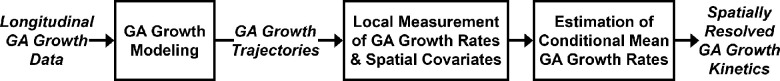
A high-level overview of the analysis approach used in this study. Methods are shown boxed, whereas data are unboxed. Details of these methods and data are provided in subsequent sections.

Although the methodologic particulars are detailed in subsequent sections, it is helpful to first outline our general approach to analyzing spatial variations of GA growth kinetics. With reference to Figure 1, our analysis involves three steps: (1) GA growth modeling, (2) local measurement of GA growth rates and corresponding spatial covariates, and (3) estimation of the conditional mean GA growth rates with respect to these covariates. At a high level, step 1 generates growth trajectories, which describe how the GA lesion grows locally, step 2 uses these trajectories to extract local growth rates and spatial covariates (e.g., margin eccentricity), and step 3 uses statistical methods to estimate average GA growth rates, conditioned on the given spatial covariates. The main contribution of this article is the development of this framework to study spatially resolved growth kinetics.

### Patient Enrolment and Selection

Patients with GA secondary to nonexudative age-related macular degeneration were enrolled in a prospective optical coherence tomography angiography (OCTA) imaging study that was approved by the Institutional Review Board of the University of Miami Miller School of Medicine. Enrolment was from June 2016 through November 2019. Informed consent was obtained from each subject. This study was performed in accordance with the tenets of the Declaration of Helsinki and complied with the Health Insurance Portability and Accountability Act of 1996.

For our present study, OCT and OCTA imaging data were extracted from the baseline visit (visit 1) and a follow-up visit (visit 2) approximately 1 year thereafter. Eyes were excluded if GA was continuous with parapapillary atrophy, if there was macular atrophy owing to a diagnosis other than nonexudative age-related macular degeneration, if there was any history of exudative macular neovascularization, or if treatment naïve, nonexudative macular neovascularization was identified by swept-source OCTA imaging. Furthermore, we required that (1) the total GA area was 2.54 mm^2^ or greater (1 disc area), (2) for multifocal lesions, at least one GA focus had an area of more than 1.25 mm^2^, and (3) the GA lesions were fully contained within the 6 mm × 6 mm field of view at both visits (see subsequent section for GA measurement and imaging details).

### OCT Imaging, Preprocessing, and Lesion Characterization

The OCT imaging protocol and preprocessing steps used in this study have been described in a previous study by our group.^10^ Briefly, eyes were imaged using a commercial swept source OCT instrument (PLEX Elite 9000; Carl Zeiss Meditec, Dublin, CA) operating at a 1050-nm central wavelength, 100-nm bandwidth, and 200-kHz A-scan rate. Full-width-at-half-maximum axial and transverse optical resolutions were 5 µm and 20 µm in tissue, respectively. Eyes were imaged over a 6 mm × 6 mm field of view using an OCTA protocol comprised of 500 A-scans per B-scan, two repeated B-scans per B-scan position, and 500 B-scan positions per volume. OCTA volumes were generated using the complex optical microangiography algorithm.^20^ Acquisitions having a signal strength of less than 7 and/or severe motion artifacts were excluded.

Visit 1 and visit 2 GA lesions were traced manually using OCT hypertransmission in en face sub–retinal pigment epithelium OCT slabs generated by projecting the OCT volume from 64 µm to 400 µm below Bruch's membrane; the presence and extent of GA was confirmed by OCT B-scan analysis, per the Classification of Atrophy Meetings criteria.^1^^,^^10^^,^^21^ The GA tracing was performed by two independent graders (Y.S. and L.W.) using commercial image analysis software (Adobe Photoshop CC; Adobe Systems, San Jose, CA) and a consensus outline was reached by the two graders. For cases in which a consensus was not reached, a senior grader (P.J.R.) acted as the adjudicator.

Visit 1 and visit 2 data were registered spatially using a second-order polynomial registration of corresponding features (retinal bifurcations) manually selected on full retinal en face OCTA projections.^22^ We opted for polynomial registration as we found that, owing to motion artifacts, affine registration was insufficiently flexible; we opted for a second-order polynomial because we found that higher order polynomials did not improve the registration quality, as subjectively assessed by retinal vasculature overlays. The position of the foveal center was approximated as the geometric center of the foveal avascular zone (FAZ), as determined by manual tracing on the visit 1 full retinal en face OCTA projections. Following prior literature,^16^ lesions were classified as foveal center point involved or foveal center point spared according to whether the foveal center was within the region of atrophy. Lesions were also classified as foveal zone involved or foveal zone spared according to whether there was any atrophy within 750 µm of the foveal center. Global lesion growth rates were characterized by the area growth rate (mm^2^/year), the square root of area growth rate (mm/year),^21^^,^^23^ and the effective radius growth rate (mm/year)^16^ (see Supplementary Material I for definitions). Note that the effective radius growth rate is related to the square root of area growth rate by a factor of $\sqrt{\pi }$. The effective radius growth rate is convenient because it has a clear physical interpretation and the same scaling as our local GA growth rate measurements, which are discussed elsewhere in this article. We report all three global growth rate metrics to facilitate comparison with prior studies.

### Estimation of GA Growth Trajectories, Growth Vectors, and Local Growth Rates

Local GA growth rates were estimated using the growth modeling framework developed in a prior study by our group.^19^ The mathematical formulation, given in Supplementary Material II, differs somewhat from our prior study, although the effects of these differences are relatively minor. Briefly, we use a biophysical GA growth model to evolve the visit 1 GA margin to the visit 2 GA margin. The GA growth model is expressed as a partial differential equation composed of two terms: a term that causes the lesion margin to expand in the direction perpendicular to its boundary (i.e., directly outward) and a term that causes concave margin segments to expand faster than convex margin segments. The rationale for these terms is discussed in our prior study.^19^

**Figure 2.**
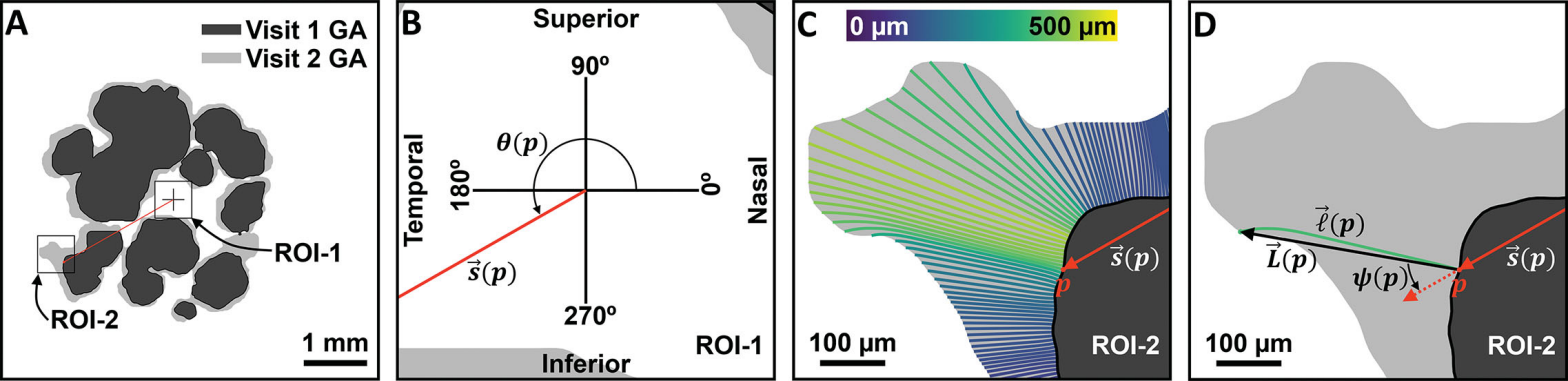
Illustration of the geometry of spatially resolved GA growth rate measurements. (**A**) Visit 1 and visit 2 GA, which were separated by approximately 1 year. Boxes indicate region of interest (ROI)-1 and ROI-2, which are enlarged in subsequent figure panels. ROI-1 contains the fovea-centered coordinate system, and ROI-2 contains a specific margin point, p. The *red line* (vector) corresponds with the position vector, $\vec{s}\left(p\right)$. (**B**) Enlargement of ROI-1. The margin angle, θ(p), of point p is defined as the counterclockwise angle that $\vec{s}\left(p\right)$ makes with the given coordinate system. (**C**) Enlargement of ROI-2, with GA growth trajectories, $\vec{\ell }$, overlaid. Growth trajectories, which connect points on the visit 1 margin to those on the visit 2 margin, are colored according to their arclength (see *color bar*). (**D**) Enlargement of ROI-2, with the growth trajectory, $\vec{\ell }\left(p\right)$, for margin point p overlaid. The geometry of the growth vector, $\vec{L}\left(p\right)$, as well as the growth angle, ψ(p), are illustrated.

For our current study, it is helpful to introduce several parameters related to the local GA growth computation (Table and Fig. 2). The growth trajectory, $\vec{\ell }$, is the spatial path tracking the modeled lesion margin as it grows from its baseline position to its follow-up position (Figs. 2C, 2D). Note that the shape of this path, which is determined both by the terms of our growth model and by the geometry of the baseline and follow-up margins, may be straight or curved. In contrast, the growth vector, $\vec{L}$, is the line segment connecting the beginning of a growth trajectory to its end (Fig. 2D); unlike the growth trajectory, it is always straight. Note that each margin point *p* has a unique growth trajectory, $\vec{\ell }\left(p\right)$, and a unique growth vector, $\vec{L}\left(p\right)$; however, margin points whose trajectories were involved in intrafocus or interfoci merging were excluded from the analysis. This exclusion is detailed in the Discussion, and in Supplementary Material V.

**Table.** Summary of Parameters and Notation Used for Quantifying GA Growth Geometry

Parameter	Description
Margin point, p	A margin point p is a specific point on the margin of the visit 1 lesion.
Growth trajectory$,\;\vec{\ell }$	The growth trajectory $\vec{\ell }\left(p\right)$ is the spatial path tracking the modeled lesion margin as it grows from its baseline position to its follow-up position (Figs. 2C, 2D). In general, a growth trajectory will have a curved, rather than straight, path.
Growth vector, $\vec{L}$	The growth vector $\vec{L}\left(p\right)$ is the vector connecting the beginning and end of the growth trajectory, $\vec{\ell }\left(p\right)$ (Fig. 2D.). Unlike $\vec{\ell }\left(p\right)$, $\vec{L}\left(p\right)$ corresponds to a straight, rather than curved, path. $\vec{L}\left(p\right)$ has units of distance (e.g., millimeters).
Local growth rate, v	The local growth rate v(p) is the estimated speed at which the lesion at margin point p is expanding. The local growth rate is computed by dividing the arclength of the growth trajectory $\vec{\ell }\left(p\right)$ by the intervisit time. v(p) has units of distance per time (e.g., millimeters per year).
Position vector, $\vec{s}$	The position vector $\vec{s}\left(p\right)$ is the vector specifying the position of margin point p relative to the fovea (Fig. 2). $\vec{s}\left(p\right)$ has units of distance (e.g., millimeters).
Margin eccentricity, r	The margin eccentricity r(p) is the distance between the margin point p and the foveal center, or, equivalently, the length of the position vector $\vec{s}\left(p\right)$. r(p) has units of distance (e.g., millimeters).
Margin angle, θ	The margin angle θ(p) is the counterclockwise angle between the position vector and the given fovea-centered coordinate system (Fig. 2B). θ(p) is specified such that margin angles of 0°, 90°, 180°, and 270° correspond to the nasal, superior, temporal, and inferior aspects of the fundus, respectively. θ(p) has units of degrees, taking values in [0°, 360°].
Growth angle, ψ	The growth angle ψ(p) describes the angle of growth relative to the foveal center and is the smallest angle between the growth vector $\vec{L}\left(p\right)$ and the position vector$\;\vec{s}\left(p\right)$ (Fig. 2D). A growth angle of 0° corresponds with growth directly away from the fovea center, and a growth angle of 180° corresponds with growth directly toward the fovea center. ψ(p) has units of degrees, taking values in [0°, 180°].

An ambiguity arises at margin points having no growth, because at these points the growth trajectory is a single point (i.e., zero length), which makes the angle of the growth vector undefined. Nevertheless, we would still like to include such points in our analysis, because they convey useful information about the GA growth pattern at that point. Thus, at margin points having no growth, we define the growth vector to be the ε-length vector, where ε is some small positive number, oriented outward and perpendicular to the margin at that point. As explained in Measuring the Spatial Configuration of GA Growth, because only the direction of the growth vector is used, the actual value of ε is inconsequential.

Finally, the local growth rate, *v*, is computed as the arclength of a growth trajectory divided by the intervisit time, and is the local analogue to typical measures of global GA growth rate. In particular, for a circular lesion undergoing isotropic growth, the local growth rate is equal to the effective radius growth rate, or $1/\sqrt{\pi }$ × the square root of area growth rate.

### Measuring the Spatial Configuration of GA Growth

In this study, we describe the spatial configuration of each GA margin using three parameters: the margin eccentricity, *r*; the margin angle, θ; and the growth angle, ψ (Table). For each point on the GA lesion margin, these parameters were measured with respect to a fovea-centered coordinate system, which was configured with one axis along the inferior–superior direction and the other axis along the temporal–nasal direction (Fig. 2). As shown in Figure 2, for a given margin point *p*, the margin eccentricity was defined as the distance of the margin point *p* from the fovea center, which is precisely equal to the length of the position vector, $\vec{s}\left(p\right)$, which specifies the position of *p* relative to the given fovea-centered coordinate system. Similarly, the margin angle, θ, was defined as the counterclockwise angle between the position vector, $\vec{s}\left(p\right)$, and the given fovea-centered coordinate system. Note that the coordinate system is oriented so that margin angles of 0°, 90°, 180°, and 270° correspond with the nasal, superior, temporal, and inferior aspects of the fundus, respectively. Note that, to allow data from right and left eyes to be analyzed jointly, left eyes were reflected about the inferior–superior axis. Finally, the growth angle, ψ, was defined as the smallest angle between the growth vector, $\vec{L}\left(p\right)$, and the position vector, $\vec{s}\left(p\right)$; that is, ψ is the arccosine of the normalized dot product of $\vec{L}\left(p\right)$ and $\vec{s}\left(p\right)$. With this convention, a growth angle of 0° corresponds with growth directly away from the fovea center, and a growth angle of 180° corresponds with growth directly toward the fovea center.

### Analysis of Spatial Variations in GA Growth Rates

For each eye, local GA growth rates, margin eccentricities, margin angles, and growth angles were measured at GA margin points distributed 6 µm apart (in arclength) along the margin. These measurements were then pooled across all study eyes and relationships between local GA growth rates and each of the covariates were independently assessed via: (1) density scatter plots, (2) box plots, and (3) Nadaraya–Watson kernel regression, a common nonparametric regression approach^24^ (Supplementary Material III). Density scatter plots, which display the relative density of the measured points falling within a particular region of the plot, were created with the ‘dscatter’ MATLAB (version 2019b; MathWorks, Inc., Natick, MA) function using default arguments.^25^ Boxplots were created using the MATLAB function ‘boxplot’, with outliers defined as those having growth rates faster than the 75th percentile by more than the 1.5 times the interquartile range, or slower than the 25th percentile by more than the 1.5 times the interquartile range. Nadaraya–Watson kernel regression was performed with subjectively chosen bandwidths of σ = 125 µm for margin eccentricities and σ = 10° for margin and growth angles. The choice of these bandwidths is considered in the Discussion.

In addition to the single-covariate analysis, a fundus growth map showing the estimated average growth rate conditioned on fundus position (i.e., conditioned on margin eccentricity and margin angle, jointly) was generated via Nadaraya–Watson kernel regression with a bandwidth of σ = 125 µm. To decrease fluctuations caused by low margin point densities, growth rate estimates at fundus positions having fewer than 250 margin points (considering margin points from all eyes) within 2σ = 250 µm were not displayed. The threshold of 250 margin points within 250 µm was subjectively chosen before the analysis by considering the spatial distribution of the lesion margins and the bandwidth of the kernel regression. We emphasize that this threshold was only applied to the fundus growth map, and not to any of the single covariate analyses.

Finally, the statistical distribution of local growth rates was descriptively investigated by (1) a histogram of the local GA growth rates and (2) a quantile–quantile plot of the observed quantiles of the local GA growth rates versus those of a fitted exponential function. The histogram was computed using the MATLAB function ‘histogram’ with bins having widths of 5 µm. The bin width was chosen as a plausible lower bound on the accuracy of GA tracing. For the quantile–quantile plot, an exponential reference function was chosen from qualitative inspection of the local growth rate histograms. The exponential function was fit using the MATLAB function ‘fitdist.’ The quantile–quantile plot was generated with the MATLAB function ‘qqplot.’

### Descriptive Statistics of Patient, Lesion, and Margin Point Characteristics

Box plots were used to summarize the statistical distributions of baseline patient ages, intervisit times, number of lesion foci at baseline, baseline lesion areas, number of margin points, area growth rates, effective radius growth rates, and square root of area growth rates. As with the assessment of spatial GA growth rate variations, boxplots were created using the MATLAB function ‘boxplot,’ with outliers defined as those having values higher than the 75th percentile by more than the 1.5 times the interquartile range, or lower than the 25th percentile by more than the 1.5 times the interquartile range.

The number of eyes and margin points used in estimating the average conditional GA growth rates were computed for each covariate (margin eccentricity, margin angle, and growth angle). The computation, which involves a sum weighted by a Gaussian kernel having a bandwidth matched to the corresponding Nadaraya–Watson kernel, is described in Supplementary Material IV.

## Results

**Figure 3.**
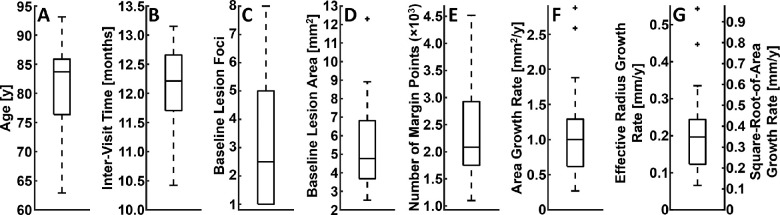
Boxplots of patient and lesion characteristics for the 38 eyes included in this study. (**A**) Baseline patient age. (**B**) Intervisit time. (**C**) Number of lesion foci at baseline. (**D**) Baseline lesion area. (**E**) Number of margin points included the analysis. (**F**) Area growth rate. (**G**) Effective radius growth rate and square root of area growth rate; note that the latter and former are related by a factor of $\sqrt{\pi }$ (see Supplementary Material I). Outliers are indicated by crosses (see Methods).

**Figure 4.**
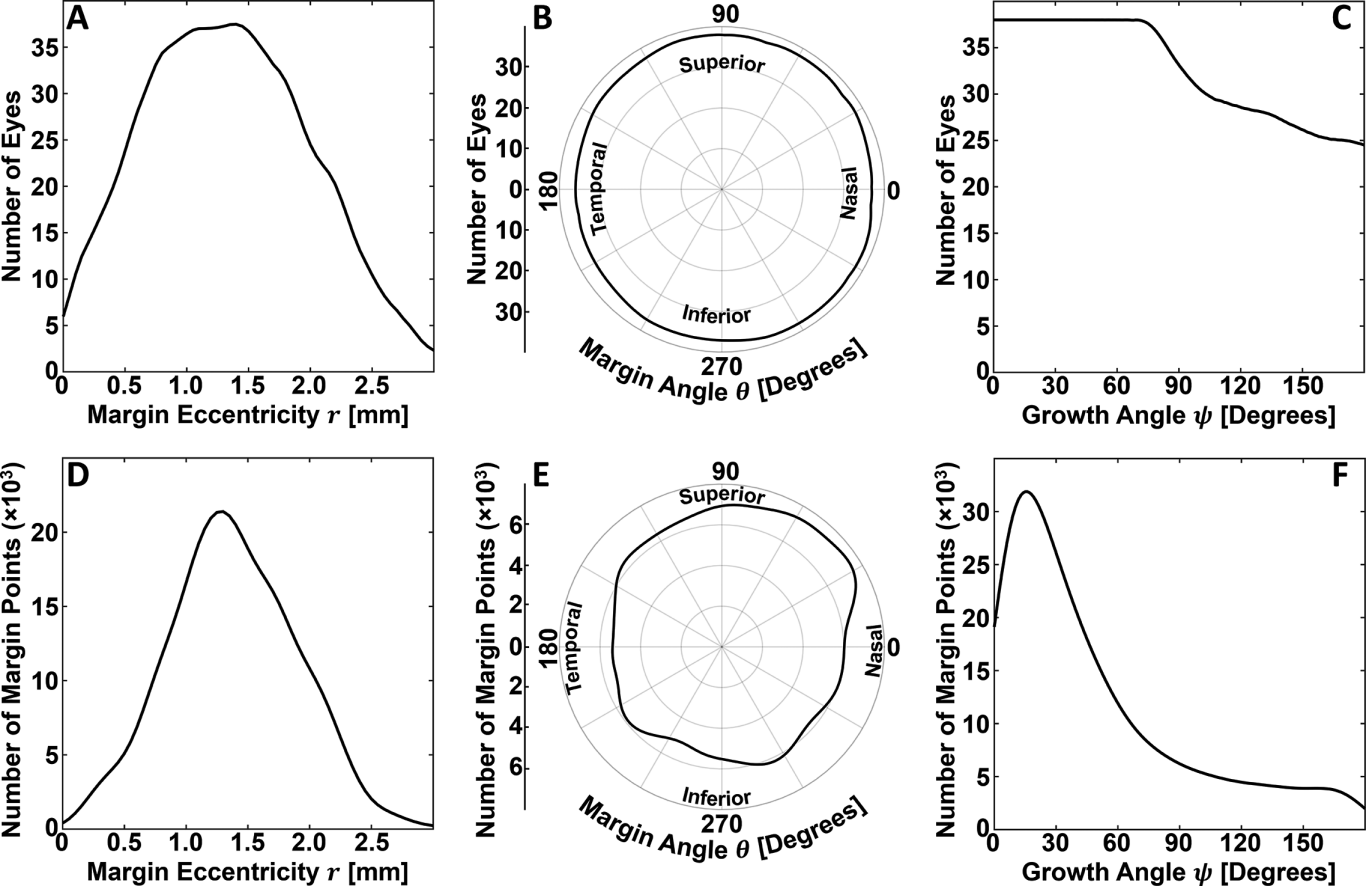
Spatial variations in the number of eyes and number of margin points used to estimate the conditional average GA growth rates. (**A**–**C**) Weighted number of eyes used to estimate average GA growth rates conditioned on margin eccentricity, margin angle, and growth angle, respectively. (**D**–**F**) Weighted number of margin points used to estimate average GA growth rates conditioned on margin eccentricity, margin angle, and growth angle, respectively. The weighting scheme and computational details are provided in Supplementary Material IV.

**Figure 5.**
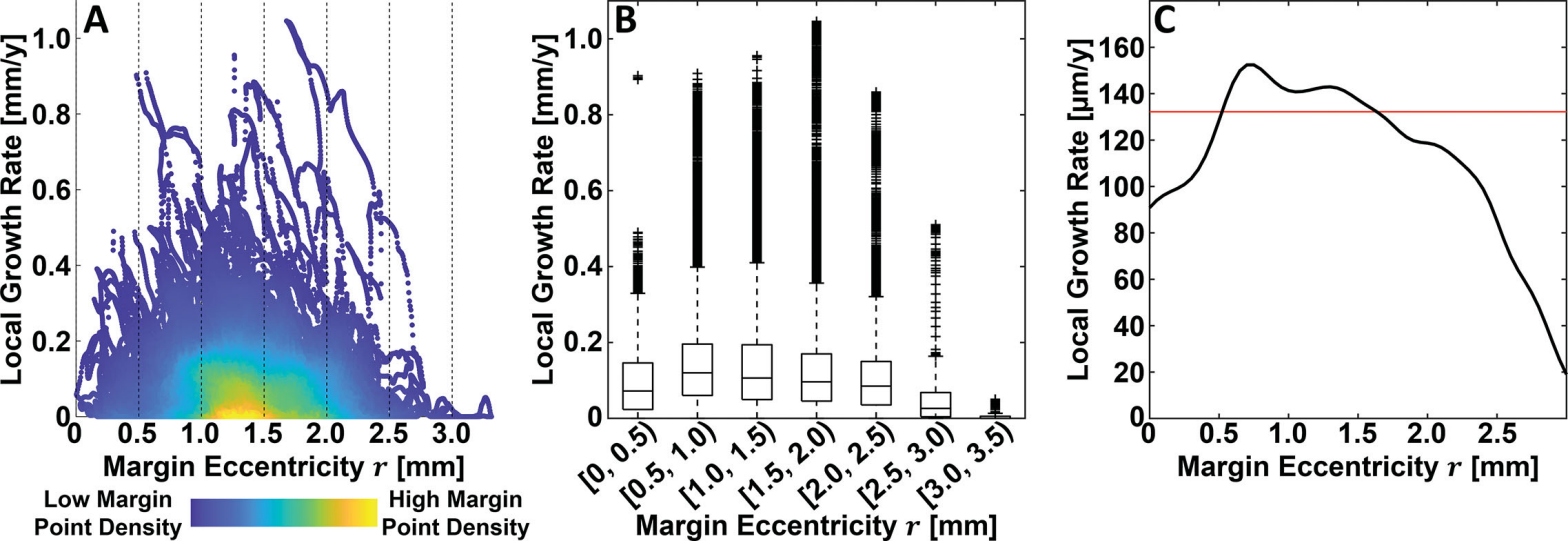
Relationship between local GA growth rate and margin eccentricity. (**A**) Density scatter plot, where colors indicate the relative density of margin point measurements (see *color bar*). (**B**) Box plots, where outliers are marked with crosses (see Methods). (**C**) Conditional mean local growth rate estimated via kernel regression (*black line*); the horizontal red line corresponds to the (unconditional) mean local GA growth rate, computed over all margin eccentricities.

**Figure 6.**
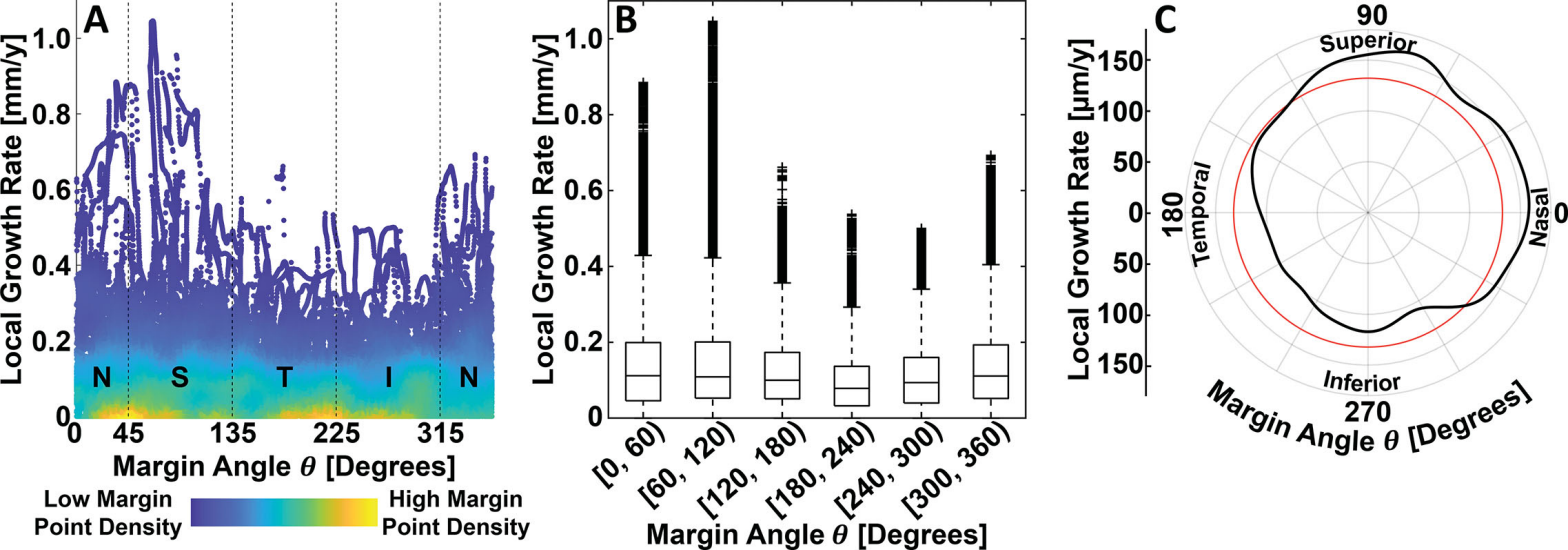
Relationship between local GA growth rate and margin angle. (**A**) Density scatter plot, where colors indicate the relative density of margin point measurements (see color bar). N = nasal; S = superior; T = temporal; and I = inferior. (**B**) Box plots, where outliers are marked with crosses (see Methods). (**C**) Conditional mean local growth rate estimated via kernel regression (*black line*), displayed on a polar plot; the circular red line corresponds to the (unconditional) mean local GA growth rate, computed over all margin angles.

**Figure 7.**
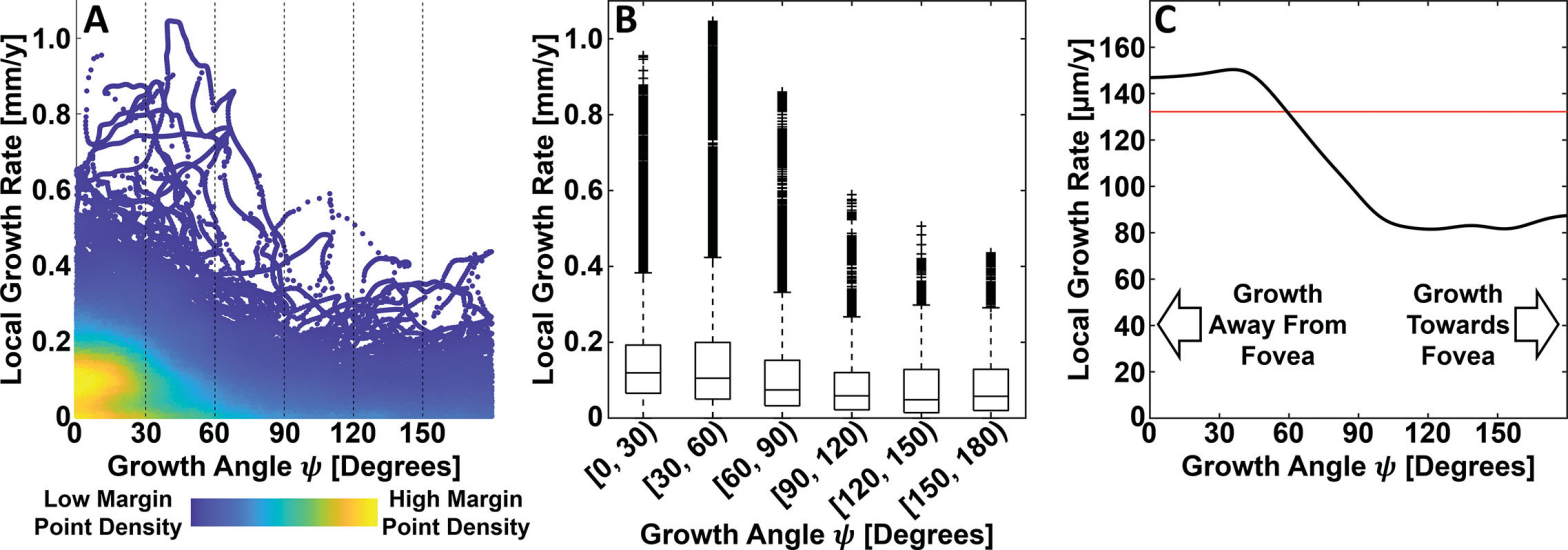
Relationship between local GA growth rate and growth angle. (**A**) Density scatter plot, where colors indicate the relative density of margin point measurements (see *color bar*). (**B**) Box plots, where outliers are marked with crosses (see Methods). (**C**) Conditional mean local growth rate estimated via kernel regression (black line); the horizontal red line corresponds to the (unconditional) mean local GA growth rate, computed overall growth angles.

**Figure 8.**
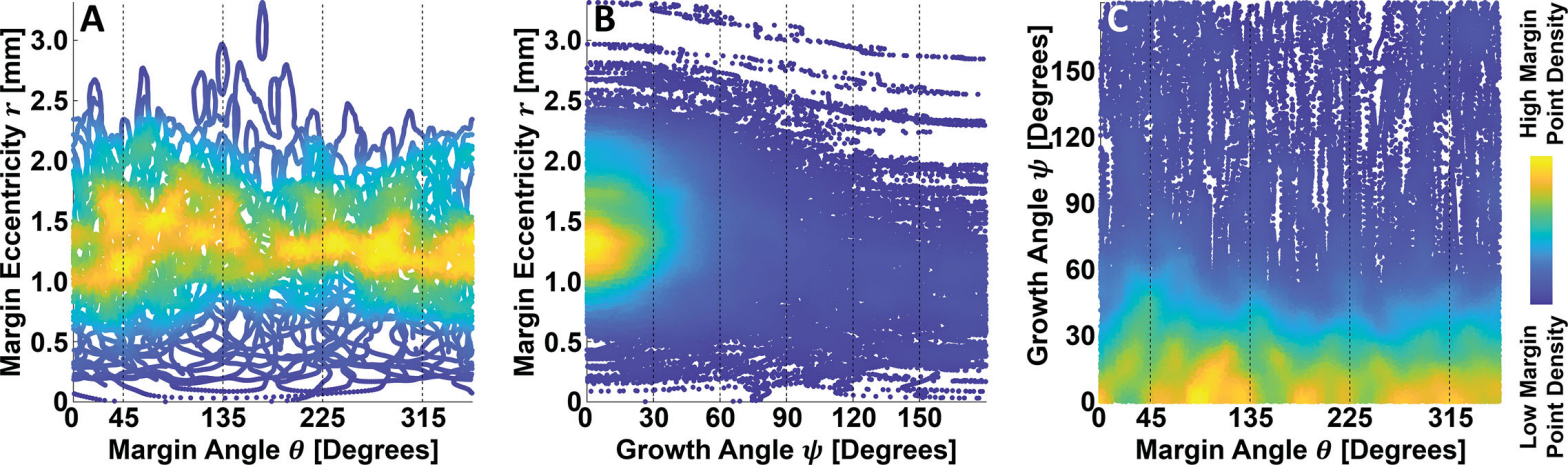
Correlations between margin eccentricity, margin angle, and growth angle. (**A**–**C**) Density scatter plots of margin eccentricity versus margin angle, margin eccentricity versus growth angle, and growth angle versus margin angle, respectively. Colors correspond with the relative density of margin points (see *color bar*, far right). Subjectively, of the three relationships, the correlation between margin eccentricity and growth angles appears most pronounced. In particular, margin points growing away from the fovea center tend to also be farther from the fovea center.

**Figure 9.**
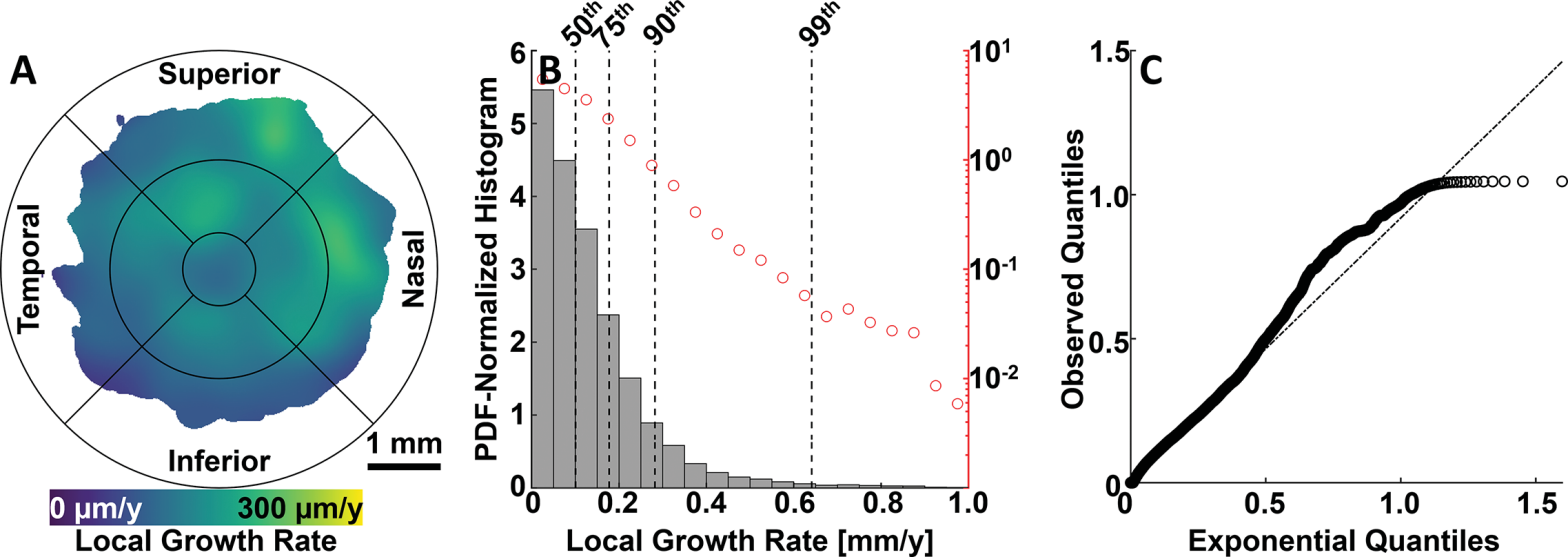
Spatial mapping and descriptive statistics of local GA growth rates. (**A**) A 6 mm × 6 mm fovea-centered fundus map of mean GA growth rates, estimated via kernel regression (see *color bar*). Regions with relatively sparse measurements are uncolored (see Methods). The Early Treatment Diabetic Retinopathy Study (ETDRS) grid is overlaid for reference. (**B**) Histogram of local GA growth rates, where histogram counts have been normalized so as to correspond to a probability density function (PDF). *Red circles* and right *y*-axis correspond with a logarithmic scale. *Dashed vertical lines* indicate the 50th, 75th, 90th, and 99th percentiles. (**C**) Quantile-quantile plot of local GA growth rates versus a fitted exponential function.

A total of 38 eyes from 27 patients were included in this study. These eyes have been used in other studies by our group.^10^ All 38 eyes (100%) had lesions that were classified as foveal zone involved and 31 eyes (82%) were classified as foveal center point involved (see Methods). A total of 94,870 margin points were modeled, 88,356 (93%) of which were included in the analysis, and 6514 (7%) of which were excluded owing to lesion merging. Patient and GA lesion characteristics are summarized in Figure 3 and Figure 4. Relationships between local GA growth rates and margin eccentricity, margin angle, and growth angle are shown in Figures 5 to 8. Fundus GA growth rate maps and statistical descriptions of local GA growth rates are presented in Figure 9.

## Discussion

The results of this study support the possibility of generating accurate, spatially resolved atlases of average GA growth rates. Such growth atlases may have several applications. First, and most immediately, assuming that the results of this study are verified in larger patient cohorts, the reported spatial trends could provide physicians and patients with additional information, albeit somewhat coarse, regarding the rate at which a lesion will expand given its location and margin configuration—for example, by considering its position on a fundus growth map, such as that shown in Figure 9A. Second, the incorporation of spatial variations in GA growth rates may help to refine predictive GA growth models, which themselves may find use in multiple applications, including in forecasting GA-related vision loss on a patient-by-patient basis, in better understanding GA pathogenesis and in selecting patients for therapeutic trials. Third, GA growth rate atlases may contribute to the understanding of GA pathophysiology, particularly if the spatial patterns of GA growth can be demonstrated to correlate with the spatial distribution of cells,^26^^,^^27^ deposits,^28^ or other entities thought to influence GA growth. And fourth, spatial variations in GA growth rates may be helpful for more accurately evaluating the effects of potential therapeutics that slow or stop GA growth. For example, if a therapeutic decreases GA growth rates by a fixed percentage, that may manifest in different absolute average decreases in GA growth rates for eyes with lesions located in the parafovea versus perifovea—a difference that could confound the accurate evaluation of the therapeutic efficacy.

Although a central aim this study was to demonstrate the applicability of our growth modelling approach, it is interesting to consider the results of our analysis in the context of prior studies. Of course, this consideration should be performed while noting the limited number of eyes (*n* = 38) in our cohort, and the limited 6 mm × 6-mm field of view. Moreover, it is important to note that, given the wide variability in lesion geometry, not all conditional growth rate estimates were computed using the same number eyes or margin points (Fig. 4). For example, the mean growth rate estimates for eccentricities in the [0 mm, 0.5 mm] and [2.5 mm, 3.0 mm] ranges were derived from fewer eyes and margin points than growth rate estimates for margin eccentricities in the (0.5 mm, 2.5 mm) range. Because it is reasonable to expect that the generalizability of a growth estimate for a particular spatial covariate (e.g., margin eccentricity) is a function of both the number of eyes and the number of margin points used to make the estimate, we should be particularly cautious when interpreting growth rate estimates made with relatively few eyes and margin points. With these caveats in mind, and also making note of our different measurement approach, the results of our study are reasonably congruent with those of prior studies.^14^^–^^16^ In particular, as in prior studies, we found that GA growth rates exhibit a hill-shaped dependency on eccentricity. In our study, we found the highest growth rates at eccentricities in the approximate range of [0.5 mm, 1.6 mm] range (Fig. 5), which agrees well with the report from Mauschitz et al.,^14^ who reported the highest median area growth rates at eccentricities in the [0.6 mm, 1.8 mm] range. Our results agree less well with those from Sayegh et al.,^15^ who report the highest mean area growth rates at eccentricates in the [1.5 mm, 3.0 mm] range, although the large grid spacing makes the extent of disagreement difficult to assess. Our results also differ somewhat from those of Shen et al.,^16^ who, in a meta-analysis that included data from Mauschitz et al. and Sayegh et al., concluded that the effective radius growth rates, which are, of the three measurement types (Supplementary Material I), the closest to our metric, were highest in the [0.6 mm, 3.5 mm] range. The results of Shen et al. are somewhat challenging to interpret in the context of our results because (1) they treat the measurements of Mauschitz et al. as being mean area measurements, when they are actually median area measurements. (2) Owing to the relatively large number of eyes in Mauschitz et al., and because Shen et al. weight the studies according to the number of eyes, the Mauschitz et al. results have substantial influence on the estimated means. However, in Mauschitz et al. the median lesion area in the [1.8 mm, 3.6 mm] range is 0.6 mm^2^, whereas in Sayegh et al., the mean lesion area in the [1.5 mm, 3.0 mm] range is substantially higher, at 2.65 mm^2^. Thus, weighting by lesion area or perimeter, rather than number of eyes, may change the Shen et al. estimates by decreasing the weighting of the Mauschitz et al. data in the [1.5 mm, 3.0 mm] range. (3) Finally, the Mauschitz et al. sector-wise (nasal, superior, temporal, and inferior) median growth rates in the [1.8 mm, 3.6 mm] range are individually all very low (≤0.03 mm^2^/year), substantially less than the pooled median in the [1.8 mm, 3.6 mm] range of 0.42 mm^2^/year. Thus, further studies are needed to understand to what extent the differences in our results are attributable to differences in measurement approaches and to what extent they are attributable to our limited cohort size, or other factors, such as our limited 6 mm × 6-mm field of view.

Our analysis of GA growth rates as a function of margin angle (Fig. 6), found moderate variations in mean GA growth rates (range, 100–160 µm/year), with increased growth rates in the superior and nasal sectors. Mauschitz et al. reported modestly increased median area growth rates in the nasal and temporal sectors; however, their observed increases were not statistically significant. Using distance-based measurements, Uji et al.^18^ found no statistically significant differences in growth *distances* as a function of margin angle.

In addition to a univariate analysis of margin eccentricity and margin angle, we also computed spatially resolved estimates of mean GA growth rates as a function of fundus position (Fig. 9A). However, because of the limited number of margin segments in some fundus regions, we were not able to provide estimates over the entire 6 mm × 6-mm field of view. Future studies of larger patient cohorts should expand the extent of this fundus growth rate map.

To our knowledge, the only quantitative study of GA growth rates as a function of growth direction is that of Lindner et al.^17^ In that study, GA growth rates were compared along the inner and outer margin segments in eyes with residual foveal islands. Owing to the particular spatial configurations of these lesions, GA growth directions along the outer margin segments were inferred to be growing away from the fovea, whereas growth directions along the inner margin segments were inferred to be growing toward the fovea. Their analysis found a 2.8 times faster square root of area growth rate along the outer margin segments (away from fovea growth direction) compared with the growth rates along the inner margin segments (toward fovea growth direction). In our analysis, average GA growth rates were approximately 1.9 times faster in segments growing away from the fovea than in segments growing toward the fovea (Fig. 7). Importantly, in Lindner et al., the examined lesion configurations were such that segments growing toward the fovea were necessarily closer to the fovea than segments growing away from the fovea. In our analysis, although lesion segments farther from the fovea were found to also be more likely to be growing away from the fovea, as discussed elsewhere in this article, this was not prespecified.

It is worth noting that there is no reason to expect, a priori, that our chosen covariates—margin eccentricity, margin angle, and growth angle—are independent of one another. Indeed, examination of the density scatter plots of Figure 8 suggests that growth angles orientated away from the margin tend to occur farther from the foveal center, which is geometrically intuitive; correlations between margin eccentricity and margin angle, and growth angle and margin angle seem to be less pronounced. Although not further investigated in this study, such correlations should be considered when interpreting the results of our study, and may be important when building statistical models of GA growth rates.

Although not a focus of our study, GA growth modeling also enables us to perform statistical analysis of the distribution of local GA growth rates (Figs. 9B–C). Our quantitative results agree with the qualitative observation that GA growth rates are neither uniform nor uniformly distributed. Indeed, in our cohort of eyes, 50% of margin points had growth rates of 0.10 mm/year or less and 99% of margin points had growth rates of 0.64 mm/year or less. In a logarithmic-linear scale (Fig. 9B), we see a relatively linear relationship between the normalized logarithmic histogram bin frequencies and GA growth rates, which is somewhat suggestive of an exponential distribution. However, when the observed quantiles are plotted against those of an exponential distribution, we note that our data differ in the tail from that of the exponential. The exponential (and gamma) distributions are theoretically plausible models of GA growth rates if we hypothesize that GA expands owing to the aggregation of many small-scale insults.^29^ Future studies with larger patient cohorts may add further insight into the statistical distribution of local GA growth rates.

**Figure 10.**
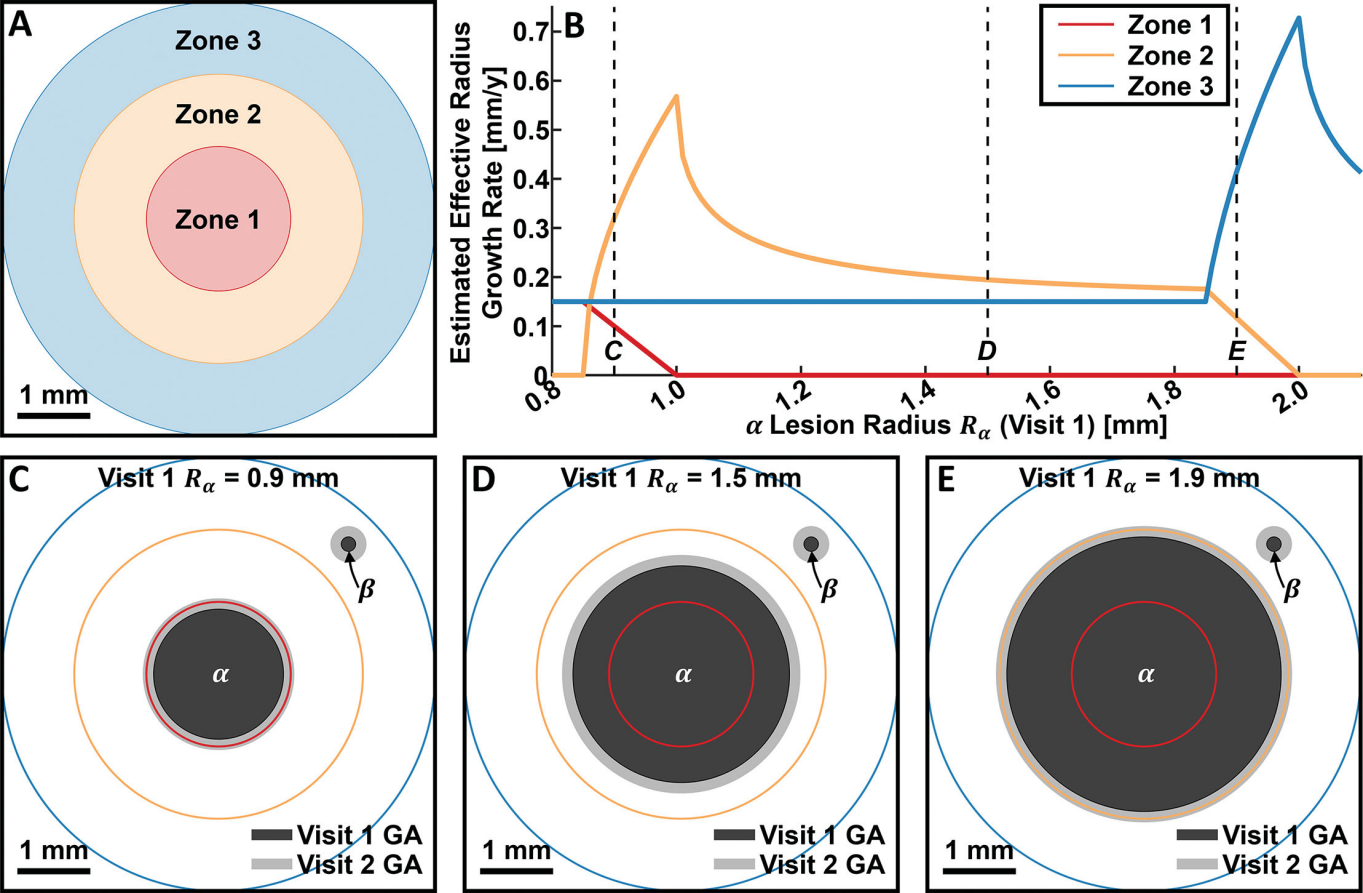
Example of simulated lesion growths, which illustrate the challenges of using grid-based approaches for spatially resolved GA growth rate measurements. In this simulated example, we consider three zones of interest: zone 1 [0 mm, 1 mm], *red*; zone 2 [1 mm, 2 mm], *orange*; and zone 3 [2 mm, 3 mm], *blue*. We suppose that there are two circular lesions: a foveal lesion (labelled α) and a perifoveal lesion (labelled β), with visit 1 (i.e., baseline) radii *R*_α_ and *R*_β_, respectively. For the simulation, we vary the foveal visit 1 lesion radius *R*_α_ between 0.8 mm and 2.1 mm, while holding the perifoveal visit 1 lesion radius *R*_β_ constant at 0.1 mm. Moreover, between visit 1 and visit 2 (1 year later), we assume that both lesions grow isotropically outward at a constant rate of 0.15 mm/year (which corresponds with an effective radius growth rate of 0.15 mm/year for each lesion, individually). (**A**) Illustration of zones of interest. (**B**) Plot of the estimated effective radius growth rates as function of *R*_α_; all other parameters are held constant. Note the large variations in estimated growth rates: zone 3 growth rate estimates are, for some configurations, more than 4.5 times the true growth rate. *Dashed vertical lines*, labelled *C–E*, refer to particular *R*_α_ values illustrated in panels C, D, and E, respectively. (**C**–**E**) Illustration of lesion configurations corresponding to the *R*_α_ values labelled *C–E* (*vertical dashed lines*) in A.

**Figure 11.**
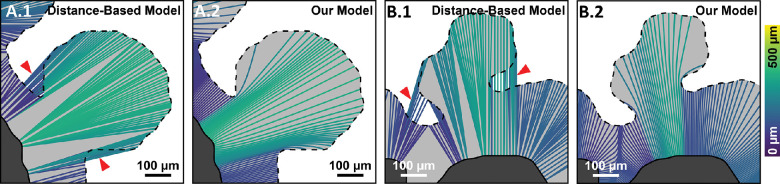
A comparison of growth trajectories from our model and those from the distance-based model.^18^ The margin segments shown are selected from lesions in this study. For all panels, *dark gray* corresponds with the visit 1 lesion and light gray with the visit 2 lesion; for clarity, the *dashed black line* outlines the visit 2 margin. Growth trajectories are colored according to length (see *color bar*, far right). (**A.1**, **A.2**) Growth trajectories corresponding to the distance-based model and to our model, respectively, for the first example margin segment. (**B.1**, **B.2**) Growth trajectories corresponding with the distance-based model and with our model, respectively, for the second example margin segment. In both example segments, the distance-based model generates nonphysical growth trajectories that intersect regions nonatrophy (*red arrowheads*).

This study demonstrates the applicability of GA growth modeling for quantifying spatial variations in GA growth rates. Compared with the existing grid-based,^14^^,^^15^ configuration-based,^17^ and distance-based^18^ strategies for spatially resolved GA growth rate measurements, we believe that our approach offers substantial advantages. First, grid-based and configuration-based techniques use either area growth rates, which are dependent on lesion perimeter,^8^^,^^9^^,^^21^^,^^23^^,^^30^ or square root transformed area growth rates,^21^^,^^23^ which assume circular lesions. In contrast, because our growth modeling approach measures growth at each margin point, it is less dependent on lesion geometry. Second, for grid-based approaches, estimated growth rates are strongly influenced by the lesion and grid geometry, which can result in both overestimates and underestimates of the true growth rate. Indeed, as illustrated in Figure 10, even simple variations in lesion geometry can lead to wide variations in the estimated growth rates. Although Figure 10 shows the effect of gridding for the case of the effective radius growth rate metric, similar effects occur when using the area growth rate metric. In contrast, our growth modeling approach is grid-free and therefore avoids these challenges. Third, because configuration-based approaches use particular lesion configurations (e.g., residual foveal islands), they are not applicable to general lesion geometries. In contrast, as mentioned elsewhere in this article, our growth modeling approach is applicable to arbitrary lesion geometries. Fourth, both grid-based and configuration-based approaches lack spatial resolution, which for grid-based approaches is determined by the grid spacing, and for configuration-based approaches is binary. Note that although the grid-based approaches can improve spatial resolution by decreasing grid spacing, this practice exacerbates the gridding artifacts. In contrast, spatial resolution in our growth modeling approach is limited only by the resolution at which the lesions can be traced, and by the spatial coverage of the margin points. Fifth, the distance-based approach relies on a closest point computation, wherein every visit 2 margin point is associated with the closest visit 1 margin point.^18^ This process implicitly models GA growth as occurring along straight line growth trajectories, which, depending on the lesion configuration, may be nonphysical and can result in growth trajectories intersecting regions of nonatrophy (Fig. 11). In contrast, our model generates physically plausible growth trajectories that never intersect regions of nonatrophy.

We note that, in addition to grid-based, configuration-based, and distance-based approaches, there have been other techniques presented in the literature that could be used to assess spatial variations in GA growth rates. For example, Pfau et al.^31^ used mixed effects logistic regression to study variations in the local likelihood of atrophy development in eyes with type 1 choroidal neovascularization. While estimating likelihoods—and not growth rates, per se—we believe that their approach is promising and could be used to study relative trends in GA growth rate variations with respect to, for example, margin eccentricity and angle (it is less clear how applicable the method is to estimating trends with respect to growth angle). However, in our present article, we have not focused on this approach because it has yet to be applied to studying spatial variations in GA growth rates in the more general context.

Despite its strengths, our growth modeling approach has a number of limitations. First, it is difficult to quantify the extent to which our GA growth model recapitulates the true GA growth dynamics, and, consequently, the accuracy with which it measures local GA growth rates. Although our estimated growth trajectories (e.g., Fig. 2, Fig. 11) seem to be plausible, further characterization of our model's accuracy, and its sensitivity to model parameters is merited. Note, however, that existing approaches to measuring GA growth rates use their own models of growth, although they may be less explicit than ours. For example, approaches that use the square root transformation model the lesion as a single circular region—a model that often does not capture the true lesion geometry well.

Another potential limitation of our GA growth modeling involves merging lesion segments. In particular, as noted in the Methods, in this study we opted to exclude margin points involved in lesion merging—owing either to merging with other segments of the same lesion focus, or with segments from a different focus. Our rationale for this exclusion is that the growth trajectory estimation becomes increasingly ill-posed after merging. Although it is possible to consider growth trajectories only up to the time when margin points merge, such an approach can greatly underestimate the true growth rate because the growth trajectories are essentially clipped—note that this is, implicitly, the approach taken by existing GA growth metrics. Excluding merging margin points avoids this limitation, but has the potential downside of introducing a selection bias as to which margin points are excluded. In particular, from a purely geometric perspective, we would expect that margins having smaller eccentricities and/or those whose growth is oriented toward the fovea would be disproportionately likely to merge, and therefore to be excluded. Indeed, this is borne out by analysis (Supplementary Material V). These spatial variations in the likelihood of margin merging, coupled with the plausible hypothesis that faster growing segments are more likely to be merge, raise the possibility of underestimating growth rates for margin segments near the fovea and/or whose growth is oriented toward the fovea. Although we do not believe that this effect is dominant in our analysis, future studies are needed to more fully investigate this potential source of bias, as well as to develop mitigation strategies, such as subsampling, to remove or control for spatial variations in the number of merging margin points.

In addition to the limitations of our growth model, there are also limitations in our dataset, our choice of spatial covariates, and our methods for estimating conditional growth rate averages. These limitations may be less relevant given our study's purpose—namely, to demonstrate the applicability of GA growth modeling for studying spatial variations in GA growth rates—but are important to mention. Related to our dataset, an important limitation, already noted, is our modest cohort size, comprising 38 eyes from 27 patients. Clearly, the results from such a small cohort have limited generalizability. Nevertheless, as discussed elsewhere in this article, our results agree reasonably well with prior studies, especially considering methodologic differences. Our use of 6 mm × 6-mm fields of view, which were selected based on available OCT/OCTA imaging technology, results in additional caveats to our growth estimates in the perifovea. Indeed, an inclusion criterion of this study is that both the visit 1 and visit 2 lesions are entirely contained within the 6 mm × 6-mm field of view. This criterion potentially excludes some GA lesions having fast growing segments close to the edges of the field of view and may therefore introduce a downward bias in perifoveal growth rate estimates. This potential bias can be addressed in future studies by using wider field of view OCT imaging. Because the Classification of Atrophy Meetings consensus group has used OCT as the basis of defining GA or complete retinal pigment epithelium and outer retinal atrophy, the use of the OCT hypertransmission defect, in conjunction with B-scan consultation, for GA lesion tracing is consistent with the current consensus recommendation and has been shown to correlate highly with autofluorescence imaging.^32^ Therefore, it is unlikely that the use of autofluorescence imaging would yield more accurate GA boundaries or different results, given that autofluorescence imaging has limitations of its own. In particular, the bright light associated with autofluorescence imaging may lead to more movement artifacts, which results in blurred lesion boundaries. Moreover, the luteal pigments can obscure GA margins within the central macula and cataracts may impact the accurate detection of GA boundaries, although these limitations have largely been resolved with green autofluorescence imaging.^33^ Even if the results differ when using autofluorescence imaging, which is unlikely, it is likely that the OCT-defined boundaries are more accurate.

Our choice of spatial covariates—namely margin eccentricity, margin angle, and growth angle—is an additional limitation. Specifically, although we chose these covariates because they were used in prior studies—which makes them well-suited for the purpose of demonstrating our framework—it is plausible that there are other metrics or coordinate frames that are advantageous. For example, instead of measuring growth angles with respect to the fovea center, measuring growth angles with respect to certain contours in the rod–cone distribution may be of interest. Nevertheless, we believe that our framework is sufficiently general so as to facilitate such modifications. We also note that our three spatial covariates were measured with respect to the fovea center, the position of which was estimated as the geometric center of the FAZ, as determined by manual tracing of full retinal OCTA projections. Because the FAZ morphology changes from eye to eye, and because the FAZ boundaries can, for some eyes and acquisitions, seem ambiguous, this method of estimation likely introduces some error. However, we do not have reason to believe that this error introduced a bias, or that this error was a substantial factor in our results.

A potential limitation in our estimation of conditional growth rate averages arises from our equal weighting of all margin points in our analysis, which causes eyes with larger lesions—and, therefore more margin points—to have a greater influence on the results than eyes with smaller lesions. Because GA growth dynamics vary among different eyes, such unweighted sampling may introduce a bias. There is potentially a related complication if different sized GA lesions exhibit different growth dynamics. In larger studies, more complex models of measurement dependency or analysis of margin point subsets may help to address these concerns. Another limitation in our estimation of conditional growth rate averages is that our kernel regression does not consider correlations within the data—most notably on the spatial level, at which neighboring margin points are more likely to have similar growths than margin points far from one another. One complication of this correlation is that standard, data-driven approaches to selecting kernel bandwidths, such as cross-validation, estimate bandwidths that are inappropriately small, thereby resulting in greater frequency variations in the estimated mean growth rates.^34^ For this reason, we eschewed data-driven approaches in selecting our kernel bandwidths and instead chose bandwidths that we believe correspond with physiologically plausible and relevant scales of variation—that is, scales on which we would expect, on the basis of clinical observation and previous growth rate studies, there to plausibly be variations in mean GA growth rates. Although more sophisticated techniques for the data-driven kernel bandwidth estimation exist,^34^ given the added complexity, the purpose of our study, and our limited cohort size, we did not pursue them. Correlations in our data also complicate the estimates of confidence intervals for the kernel regression estimates, which is why we did not report confidence intervals in our analysis. In future studies with larger datasets, we hope to address these limitations.

## Conclusions

In this study, we introduced a GA growth modeling framework for quantitatively analyzing spatial variations in GA growth rates. Demonstrating the applicability of our approach on 38 eyes, we found reasonable agreement between our results and those of prior studies. Based on the results of this pilot study, we believe that our approach is well-suited to apply to larger GA datasets with the aim of constructing accurate, spatially resolved atlases of average GA growth rates across the fundus. Although demonstrated on OCT data, our framework should be equally applicable to other imaging modalities, including color fundus photography and fundus autofluorescence.

## Supplementary Material

Supplement 1

Supplement 2

Supplement 3

Supplement 4

Supplement 5

## References

[1] Sadda SR, Guymer R, Holz FG, et al. Consensus definition for atrophy associated with age-related macular degeneration on OCT: classification of atrophy report 3. *Ophthalmology*. 2018; 125: 537–548. 29103793 10.1016/j.ophtha.2017.09.028 PMC11366072

[2] Bhutto I, Lutty G. Understanding age-related macular degeneration (AMD): relationships between the photoreceptor/retinal pigment epithelium/Bruch's membrane/choriocapillaris complex. *Mol Aspects Med*. 2012; 33: 295–317. 22542780 10.1016/j.mam.2012.04.005 PMC3392421

[3] McLeod DS, Grebe R, Bhutto I, Merges C, Baba T, Lutty GA. Relationship between RPE and choriocapillaris in age-related macular degeneration. *Invest Ophthalmol Vis Sci*. 2009; 50: 4982–4991. 19357355 10.1167/iovs.09-3639 PMC4829357

[4] Bird AC, Phillips RL, Hageman GS. Geographic atrophy: a histopathological assessment. *JAMA Ophthalmol*. 2014; 132: 338–345. 24626824 10.1001/jamaophthalmol.2013.5799 PMC4853921

[5] Holz FG, Strauss EC, Schmitz-Valckenberg S, van Lookeren Campagne M. Geographic atrophy: clinical features and potential therapeutic approaches. *Ophthalmology.* 2014; 121: 1079–1091. 24433969 10.1016/j.ophtha.2013.11.023

[6] Keenan TD, Agrón E, Domalpally A, et al. Progression of geographic atrophy in age-related macular degeneration: AREDS2 report number 16. *Ophthalmology*. 2018; 125: 1913–1928. 30060980 10.1016/j.ophtha.2018.05.028 PMC6246813

[7] Fleckenstein M, Mitchell P, Freund KB, et al. The Progression of geographic atrophy secondary to age-related macular degeneration. *Ophthalmology*. 2018; 125: 369–390. 29110945 10.1016/j.ophtha.2017.08.038

[8] Pfau M, Lindner M, Goerdt L, et al. Prognostic value of shape-descriptive factors for the progression of geographic atrophy secondary to age-related macular degeneration. *Retina*. 2019; 39: 1527–1540. 29781974 10.1097/IAE.0000000000002206

[9] Domalpally A, Danis RP, White J, et al. Circularity index as a risk factor for progression of geographic atrophy. *Ophthalmology*. 2013; 120: 2666–2671. 24206616 10.1016/j.ophtha.2013.07.047

[10] Thulliez M, Zhang Q, Shi Y, et al. Correlations between choriocapillaris flow deficits around geographic atrophy and enlargement rates based on swept-source OCT imaging. *Ophthalmol Retina*. 2019; 3: 478–488. 31174669 10.1016/j.oret.2019.01.024 PMC11402513

[11] Nassisi M, Shi Y, Fan W, et al. Choriocapillaris impairment around the atrophic lesions in patients with geographic atrophy: a swept-source optical coherence tomography angiography study. *Br J Ophthalmol*. 2019; 103: 911. 30131381 10.1136/bjophthalmol-2018-312643

[12] Alagorie AR, Nassisi M, Verma A, et al. Relationship between proximity of choriocapillaris flow deficits and enlargement rate of geographic atrophy. *Graefes Arch Clin Exp Ophthalmol*. 2020; 258: 995–1003. 32043168 10.1007/s00417-020-04615-w

[13] Holz FG, Bindewald-Wittich A, Fleckenstein M, Dreyhaupt J, Scholl HPN, Schmitz-Valckenberg S. Progression of geographic atrophy and impact of fundus autofluorescence patterns in age-related macular degeneration. *Am J Ophthalmol*. 2007; 143: 463–472.e462. 17239336 10.1016/j.ajo.2006.11.041

[14] Mauschitz MM, Fonseca S, Chang P, et al. Topography of geographic atrophy in age-related macular degeneration. *Invest Ophthalmol Vis Sci*. 2012; 53: 4932–4939. 22661483 10.1167/iovs.12-9711

[15] Sayegh RG, Sacu S, Dunavölgyi R, et al. Geographic atrophy and foveal-sparing changes related to visual acuity in patients with dry age-related macular degeneration over time. *Am J Ophthalmol*. 2017; 179: 118–128. 28385474 10.1016/j.ajo.2017.03.031

[16] Shen LL, Sun M, Khetpal S, Grossetta Nardini HK, Del Priore LV. Topographic variation of the growth rate of geographic atrophy in nonexudative age-related macular degeneration: a systematic review and meta-analysis. *Invest Ophthalmol Vis Sci*. 2020; 61: 2. 10.1167/iovs.61.1.2 PMC7205189 31995152

[17] Lindner M, Böker A, Mauschitz MM, et al. Directional kinetics of geographic atrophy progression in age-related macular degeneration with foveal sparing. *Ophthalmology*. 2015; 122: 1356–1365. 25972258 10.1016/j.ophtha.2015.03.027

[18] Uji A, Nittala MG, Hariri A, Velaga SB, Sadda SR. Directional kinetics analysis of the progression of geographic atrophy. *Graefes Arch Clin Exp Ophthalmol*. 2019; 257: 1679–1685. 31147841 10.1007/s00417-019-04368-1

[19] Moult EM, Alibhai AY, Lee B, et al. A framework for multiscale quantitation of relationships between choriocapillaris flow impairment and geographic atrophy growth. *Am. J. Ophthalmol*. 2019; 214: 172–187 31843474 10.1016/j.ajo.2019.12.006 PMC7951042

[20] An L, Wang RK. In vivo volumetric imaging of vascular perfusion within human retina and choroids with optical micro-angiography. *Opt Express*. 2008; 16: 11438–11452. 18648464 10.1364/oe.16.011438

[21] Yehoshua Z, Rosenfeld PJ, Gregori G, et al. Progression of geographic atrophy in age-related macular degeneration imaged with spectral domain optical coherence tomography. *Ophthalmology*. 2011; 118: 679–686. 21035861 10.1016/j.ophtha.2010.08.018 PMC3070862

[22] Goshtasby A. Image registration by local approximation methods. *Image Vision Comput*. 1988; 6: 255–261.

[23] Feuer WJ, Yehoshua Z, Gregori G, et al. Square root transformation of geographic atrophy area measurements to eliminate dependence of growth rates on baseline lesion measurements: a reanalysis of age-related eye disease study report No. 26. *JAMA Ophthalmol*. 2013; 131: 110–111. 23307222 10.1001/jamaophthalmol.2013.572 PMC11551521

[24] Härdle W, Müller M, Sperlich S, Werwatz A. *Nonparametric and Semiparametric Models*. Berlin, Heidelberg: Springer-Verlag; 2004.

[25] Henson R. Flow Cytometry Data Reader and Visualization MATLAB Central File Exchange. Available at: https://www.mathworks.com/matlabcentral/fileexchange/8430-flow-cytometry-data-reader-and-visualization. Accessed January 25, 2021.

[26] Curcio CA, Medeiros NE, Millican CL. Photoreceptor loss in age-related macular degeneration. *Invest Ophthalmol Vis Sci*. 1996; 37: 1236–1249. 8641827

[27] Owsley C, Jackson GR, Cideciyan AV, et al. Psychophysical evidence for rod vulnerability in age-related macular degeneration. *Invest Ophthalmol Vis Sci*. 2000; 41: 267–273. 10634630

[28] Li M, Dolz-Marco R, Huisingh C, et al. Clinicopathologic correlation of geographic atrophy secondary to age-related macular degeneration. *Retina*. 2019; 39: 802–816. 30839495 10.1097/IAE.0000000000002461 PMC6445604

[29] Frank SA. The common patterns of nature. *J Evol Biol*. 2009; 22: 1563–1585. 19538344 10.1111/j.1420-9101.2009.01775.x PMC2824446

[30] Shen LL, Sun M, Ahluwalia A, Young BK, Park MM, Del Priore LV. Geographic atrophy growth is strongly related to lesion perimeter: unifying effects of lesion area, number, and circularity on growth. *Ophthalmol Retina*. 2020 Dec 9 [Epub ahead of print]. 10.1016/j.oret.2020.12.002 33307218

[31] Pfau M, Möller PT, Künzel SH, et al. Type 1 choroidal neovascularization is associated with reduced localized progression of atrophy in age-related macular degeneration. *Ophthalmol Retina*. 2020; 4: 238–248. 31753808 10.1016/j.oret.2019.09.016

[32] Yehoshua Z, de Amorim Garcia Filho CA, Nunes RP, et al. Comparison of geographic atrophy growth rates using different imaging modalities in the COMPLETE study. *Ophthalmic Surg Lasers Imaging Retina*. 2015; 46: 413–422. 25970861 10.3928/23258160-20150422-03

[33] Pfau M, Goerdt L, Schmitz-Valckenberg S, et al. Green-light autofluorescence versus combined blue-light autofluorescence and near-infrared reflectance imaging in geographic atrophy secondary to age-related macular degeneration. *Invest Ophthalmol Vis Sci*. 2017; 58: BIO121–BIO130. 28632841 10.1167/iovs.17-21764

[34] De Brabanter K, De Brabanter J, Suykens JA, De Moor B. Kernel Regression in the Presence of Correlated Errors. *J Mach Learn Res*. 2011; 12: 1955–1976.

