# Supplementary material for: Growth Modeling for Quantitative, Spatially Resolved Geographic Atrophy Lesion Kinetics
Source: Transl Vis Sci Technol. 2021 Jun 22;10(7):26. doi: 10.1167/tvst.10.7.26 (PMC8237082; doi:10.1167/tvst.10.7.26)
Supplement: Supplement 1 [file tvst-10-7-26_s001.pdf]

Supplementary Material I: Global Geographic Atrophy Growth Rate Metrics

In this study, we report values for three metrics of global geographic atrophy (GA) growth rate. The first two metrics, which are commonly reported in the literature, are the *area growth rate* [mm^2/year] and the *square-root-of-area growth rate* [mm/year],[1, 2] defined as:

$$\text{Area Growth Rate} = \frac{\text{Visit 2 Lesion Area} - \text{Visit 1 Lesion Area}}{\text{Inter-Visit Time}}$$

$$\text{Square-Root-of-Area Growth Rate} = \frac{\sqrt{\text{Visit 2 Lesion Area}} - \sqrt{\text{Visit 1 Lesion Area}}}{\text{Inter-Visit Time}}$$

The third metric is the *effective radius growth rate* [mm/year], used in Shen et al.,[3] which is related to the square-root-of-area growth rate by a factor of $\sqrt{\pi}$:

$$\text{Effective Radius Growth Rate} = \frac{\text{Square-Root-of-Area Growth Rate}}{\sqrt{\pi}}$$

1. Yehoshua Z, Rosenfeld PJ, Gregori G, et al. Progression of Geographic Atrophy in Age-Related Macular Degeneration Imaged with Spectral Domain Optical Coherence Tomography. *Ophthalmology* 2011;118:679-686.
2. Feuer WJ, Yehoshua Z, Gregori G, et al. Square Root Transformation of Geographic Atrophy Area Measurements to Eliminate Dependence of Growth Rates on Baseline Lesion Measurements: A Reanalysis of Age-Related Eye Disease Study Report No. 26. *JAMA Ophthalmology* 2013;131:110-111.
3. Shen LL, Sun M, Khetpal S, Grossetta Nardini HK, Del Priore LV. Topographic Variation of the Growth Rate of Geographic Atrophy in Nonexudative Age-Related Macular Degeneration: A Systematic Review and Meta-analysis. *Investigative Ophthalmology & Visual Science* 2020;61:2-2.
