# Supplementary material for: Growth Modeling for Quantitative, Spatially Resolved Geographic Atrophy Lesion Kinetics
Source: Transl Vis Sci Technol. 2021 Jun 22;10(7):26. doi: 10.1167/tvst.10.7.26 (PMC8237082; doi:10.1167/tvst.10.7.26)
Supplement: Supplement 2 [file tvst-10-7-26_s002.pdf]

Supplementary Material II: Computation of Local GA Growth Rates

In this study, we computed local GA growth rates following the approach previously proposed by our group.[1] In this approach, GA growth is treated as an interface propagation problem, wherein the lesion margin expands according to a specified partial differential equation. Specifically, with $\Omega \subset \mathbb{R}^2$ the image domain, let $G(t) \subset \Omega$ be the GA area at time t and $\partial G(t)$ be its corresponding margin. To accommodate lesion merging (i.e., merging of margin segments of the same lesion focus and/or merging of margin segments of different lesion foci), we utilize the level set method,[2] whereby the GA margin is represented as the zero level set of a higher dimensional function ϕ. In particular, $G(t) = \{\phi(\boldsymbol{x}, t) \leq 0\}$ and $\partial G(t) = \{\phi(\boldsymbol{x}, t) = 0\}$, with $\boldsymbol{x} \in \Omega$. The evolution of ϕ is governed by the partial differential equation:

$$\partial_t \phi(\boldsymbol{x}, t) + F(\phi, \boldsymbol{x}, t)||\nabla \phi(\boldsymbol{x}, t)|| = 0 \tag{1}$$

where F describes the forces driving GA expansion. In this study we set:

$$F(\phi, \boldsymbol{x}, t) = \alpha - \beta\kappa(\phi) \tag{2}$$

where α and β are positive constants and κ is the curvature. The same model parameter values of $\alpha = 1$ and $\beta = 0.75$ that were used in our previous study[1] were used in the current study. As described in Moult et al.,[1] the curvature term causes concave margin segments to expand more rapidly than convex segments. Let t_b be the time of the baseline visit and t_f be the time of the follow-up visit. For any given visit pair, we measure $\partial G(t_b)$ and $\partial G(t_f)$, which serve as our boundary conditions. In terms of the level set function, we require:

$$\{\phi(\boldsymbol{x}, t_b) = 0\} = \partial G(t_b) \quad \text{and} \quad \{\phi(\boldsymbol{x}, t_f) = 0\} = \partial G(t_f) \tag{3}$$

To satisfy these constraints we define the following signed distance functions:

$$\phi_b(\boldsymbol{x}) \equiv \begin{cases} -d\big(\boldsymbol{x}, \partial G(t_b)\big) & \text{if } \boldsymbol{x} \in G(t_b) \\ +d\big(\boldsymbol{x}, \partial G(t_b)\big) & \text{if } \boldsymbol{x} \in G^c(t_b) \end{cases} \text{ and } \phi_f(\boldsymbol{x}) \equiv \begin{cases} -d(\boldsymbol{x}, \partial G(t_f)) & \text{if } \boldsymbol{x} \in G(t_f) \\ +d(\boldsymbol{x}, \partial G(t_f)) & \text{if } \boldsymbol{x} \in G^c(t_f) \end{cases} \tag{4}$$

where d is the Euclidean distance function, and G^c is the complement of G. With this, we set the boundary conditions on ϕ as:

$$\phi(\boldsymbol{x}, t_b) = \phi_b(\boldsymbol{x}) \quad \text{and} \quad \phi(\mathbf{x}, t_f) = \phi_f(\boldsymbol{x}) \tag{5}$$

During the evolution of ϕ we ensure that the second condition of Eq. 5 is satisfied by enforcing

$$\phi_f(\boldsymbol{x}) \leq \phi(\boldsymbol{x}, t) \quad \forall t \tag{6}$$

after every iteration. Equation 2 was solved numerically using the toolset developed by Mitchell.[3] With Eq. 2 solved, for each position $\boldsymbol{x}_b \in \partial G(t_b)$ we constructed a growth trajectory $\gamma(\boldsymbol{x}_b, t)$ via:

$$\frac{d}{dt}\gamma(\boldsymbol{x}_b, t) = -\partial_t\phi(\boldsymbol{x}, t)\frac{\nabla\phi(\boldsymbol{x}, t)}{\|\nabla\phi(\boldsymbol{x}, t)\|} \tag{7}$$

on the time interval $t \in [t_b, t_f]$ subject to the initial condition that $\gamma(\boldsymbol{x}_b, t_b) = \boldsymbol{x}_b$. As in our previous work, growth trajectories that merged/collided with other growth trajectories were excluded. The local growth distance, $\Gamma(\boldsymbol{x}_b)$, was computed as:

$$\Gamma(\boldsymbol{x}_b) = \int_{t_b}^{t_f} \|\dot{\gamma}(\boldsymbol{x}_b, t)\| dt \tag{8}$$

Finally, the local growth rate associated with position $\boldsymbol{x}_b$ was computed by dividing $\Gamma(\boldsymbol{x}_b)$ by the inter-visit time, $t_f - t_b$.

1. Moult EM, Alibhai AY, Lee B, et al. A Framework for Multiscale Quantitation of Relationships Between Choriocapillaris Flow Impairment and Geographic Atrophy Growth. *American Journal of Ophthalmology* 2019.
2. Osher S, Sethian JA. Fronts propagating with curvature-dependent speed: Algorithms based on Hamilton-Jacobi formulations. *Journal of Computational Physics* 1988;79:12-49.
3. Mitchell IM. The Flexible, Extensible and Efficient Toolbox of Level Set Methods. *Journal of Scientific Computing* 2008;35:300-329.
