# Supplementary material for: Growth Modeling for Quantitative, Spatially Resolved Geographic Atrophy Lesion Kinetics
Source: Transl Vis Sci Technol. 2021 Jun 22;10(7):26. doi: 10.1167/tvst.10.7.26 (PMC8237082; doi:10.1167/tvst.10.7.26)
Supplement: Supplement 3 [file tvst-10-7-26_s003.pdf]

Supplementary Material III: Kernel Regression of Local GA Growth Rates

In this study, we estimated the mean local geographic atrophy (GA) growth rate conditioned on margin eccentricity, margin angle, growth angle, and fundus position (see *Methods* and Figure 5.C, 6.C, 7.C, and 7.A). We performed this estimation using Nadaraya-Watson kernel regression with a Gaussian kernel:[1]

$$E[v|x] = \frac{\sum_{i=1}^{n_p} v_i K_h(\|x_i - x\|)}{\sum_{i=1}^{n_p} K_h(\|x_i - x\|)} \tag{1}$$

where v is the local growth rate, x is the covariate of interest (i.e., margin eccentricity, $x = r$; margin angle, $x = \theta$; growth angle, $x = \psi$; or fundus position, $x = (r, \theta)$), (v_i, x_i) are the *i*-th paired measurements, n_p is the number of margin points included in the analysis (in this study n_p= 88,356), ||·|| is the distance function, and K_h is the Gaussian kernel defined as:

$$K_h(y) = \frac{1}{\sqrt{2\pi}h} \exp\left(\frac{-y^2}{2h^2}\right) \tag{2}$$

where h is the kernel bandwidth. For estimation conditioned on the margin eccentricity, growth angle, and fundus position, ||·|| was taken to be the standard Euclidean norm. For estimation conditioned on the margin angle, ||·|| was taken to be the Euclidean norm wrapped into [0°, 180°].

1. Härdle W, Müller M, Sperlich S, Werwatz A. *Nonparametric and Semiparametric Models*: Springer-Verlag Berlin Heidelberg; 2004.
