# Supplementary material for: Growth Modeling for Quantitative, Spatially Resolved Geographic Atrophy Lesion Kinetics
Source: Transl Vis Sci Technol. 2021 Jun 22;10(7):26. doi: 10.1167/tvst.10.7.26 (PMC8237082; doi:10.1167/tvst.10.7.26)
Supplement: Supplement 4 [file tvst-10-7-26_s004.pdf]

Supplementary Material IV: Spatial Variations in the Number of Eyes and Margin Points

As a measure of the number of eyes that contribute to a conditional mean geographic atrophy (GA) growth rate estimate, we computed:

$$N_{e|x}(x) = \frac{\sum_{i=1}^{n_e} K_h\left(\min_{x_j \in X^{(i)}} \|x_j - x\|\right)}{K_h\left(\min_i \min_{x_j \in X^{(i)}} \|x_j - x\|\right)} \tag{1}$$

where x is the covariate of interest (i.e., margin eccentricity, $x = r;$ margin angle, $x = \theta$; or growth angle, $x = \psi$), $N_{e|x}$ is the number of eyes contributing to the growth rate measurement conditioned on x, n_e is the number of eyes in the study (in our study n_e = 38), $X^{(i)}$ is the set of covariate values from the *i*-th eye ($X^{(i)} = R^{(i)}$, $X^{(i)} = \Theta^{(i)}$, or $X^{(i)} = \Psi^{(i)}$), and ||·|| and K_h are the distance function and Gaussian kernel defined in *Supplementary Material III*. The bandwidths h were matched to those specified in *Supplementary Material III*.

As a measure of the number of margin points contributing to a conditional mean GA growth rate estimate, we computed:

$$N_{p|x}(x) = \frac{\sum_{i=1}^{n_p} K_h(\|x_i - x\|)}{K_h\left(\min_i \|x_i - x\|\right)} \tag{2}$$

where x is the covariate of interest, $N_{p|x}$ is the number of margin points contributing to the growth rate measurement conditioned on x, n_p is the number of margin points included in the analysis (in this study n_p= 88,356), and ||·|| and K_h are the distance function and Gaussian kernel defined in *Supplementary Material III*. Again, the bandwidths h were matched to those specified in *Supplementary Material III*.
