# Supplementary material for: Growth Modeling for Quantitative, Spatially Resolved Geographic Atrophy Lesion Kinetics
Source: Transl Vis Sci Technol. 2021 Jun 22;10(7):26. doi: 10.1167/tvst.10.7.26 (PMC8237082; doi:10.1167/tvst.10.7.26)
Supplement: Supplement 5 [file tvst-10-7-26_s005.pdf]

Supplementary Material V: Spatial Variations in the Probability of Margin Merging

In this supplementary material we consider the spatial distribution of *merging* margin points—that is margin points involved in lesion merging, either with other segments of the same lesion focus, or with segments from a different lesion focus. As noted in the text, because we excluded such points from our analysis, a relevant question becomes: *are margin points equally likely to merge for different margin eccentricities, margin angles, and/or growth angles?* To this end, consider the experiment of randomly selecting a margin point p from the set of *all* margin points (i.e., both merging and non-merging; in this study, there were 94,870 margin points, 6,514 of which were merging). For this experiment, let r be the random variable corresponding to the eccentricity of p, and let $m \in \{0,1\}$ be the random variable corresponding to whether p is a merging point ($m = 1$) or a non-merging point ($m = 0$). Furthermore, let $f_r(r)$ be the probability density function (PDF) of r and let $f_{r|m}(r|m)$ be the conditional PDF of r given m. For our purposes, a margin point being equally likely to merge at all margin eccentricities corresponds to the statement that r and m are statistically independent, or $f_{r|m}(r|m) = f_r(r)$. Thus, we can gain insight into the dependency between margin merging and margin eccentricity by comparing the plot of $f_{r|m}(r|m = 1)$ to $f_r(r)$: if lesion merging is independent of margin eccentricity, these plots would be identical (in the ideal case) or similar (in the experimental case). Note that the same reasoning is applicable to dependencies on margin angle, θ, and growth angle, ψ.

Using this approach, Figure S1 shows plots of $\hat{f}_x(x)$ and $\hat{f}_{x|m}(x|m = 1)$, the estimated PDFs of $f_x(x)$ and $f_{x|m}(x|m = 1)$, for margin eccentricity ($x = r$), margin angle ($x = \theta$), and growth angle ($x = \psi$). PDFs were estimated via kernel density estimation using the 'ksdensity' MATLAB function. Specifically, all PDFs were estimated using Gaussian kernels with bandwidths equal to those used for the Nadaraya-Watson kernel regression described in *Supplementary Material III*: σ = 125 µm for $\hat{f}_r(r)$ and $\hat{f}_{r|m}(r|m = 1)$; and σ = 10° for $\hat{f}_\theta(\theta)$, $\hat{f}_{\theta|m}(\theta|m = 1)$, $\hat{f}_\psi(\psi)$, and $\hat{f}_{\psi|m}(\psi|m = 1)$.

With reference to Figure S1.A, note that $\hat{f}_{r|m}(r|m=1)$ has greater probability density than $\hat{f}_r(r)$ for margin points closer to the fovea. This suggests that margin points closer to the fovea are more likely to merge than those farther from the fovea, which agrees with physical intuition. Similarly, with reference to Figure S1.C, note that $\hat{f}_{\theta|m}(\theta|m=1)$ has greater probability density than $\hat{f}_\theta(\theta)$ for margin points growing towards the fovea. Again, this suggests that margin points growing towards the fovea are more likely to merge than margin points growing away from the fovea. Thus, if merging margin points are also more likely to have higher growth rates (a physically intuitive, but difficult-to-test hypothesis), our growth rate estimates for margin points close to and/or growing towards the fovea may underestimate the true growth rates. Thus, spatial variations in the likelihood of lesion merging could plausibly be responsible for some portion of our finding that growth rates of margin points close to and/or growing towards the fovea are relatively slower than growth rates of margin points far from and/or growing away from the fovea (Figure 5, 7). In contrast, Figure S1.B suggests that margin points in the superior and nasal aspects are more likely to merge. If we again suppose that faster growing segments are more likely to merge, it follows that our growth rate estimates in the superior and nasal aspects may underestimate the true growth rates. However, since our growth rate estimates in the superior and nasal aspects were greater than those in the inferior and temporal aspects (Figure 5), this suggests that any (downward) bias due to preferential lesion merging is of smaller magnitude than the growth rate variations not attributable to lesion merging. In summary, while there *are* spatial variations in the probability of margin merging, their impact on our results is unclear, but merits investigation in future studies.

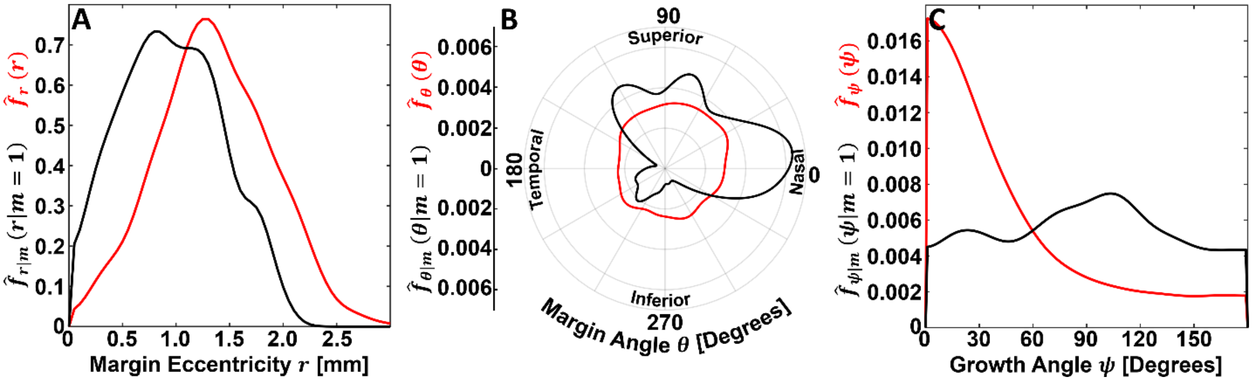

Figure S1. Analysis of spatial variations in the probability of margin merging. (A) The black curve corresponds to the estimated conditional probability density function (PDF) of margin eccentricity, $\hat{f}_{r|m}(r|m=1)$, conditioned on the event of margin merging. The red curve corresponds to the estimated PDF of margin eccentricity, $\hat{f}_r(r)$, which also corresponds to the conditional PDF under the assumption that r and m are independent. (B-C) Analogous plots for margin angle, θ, and growth angle, ψ, respectively.
